# Primary central nervous system diffuse large B‐cell lymphoma masqueraded as Bing‐Neel syndrome: Steps in management and review of future directions

**DOI:** 10.1002/ccr3.5113

**Published:** 2021-12-06

**Authors:** Lukas Delasos, Deep Phachu, Nishka Shetty, Melissa Sepulveda‐Ramos, James Vredenburgh

**Affiliations:** ^1^ Department of Internal Medicine University of Connecticut Health Center Farmington Connecticut USA; ^2^ Department of Hematopathology Hartford Hospital Hartford Connecticut USA; ^3^ Department of Hematology and Oncology Smilow Cancer Hospital at St. Francis Hartford Connecticut USA

**Keywords:** Bing‐Neel syndrome, central nervous system lymphomas, hematology, oncology, Waldenstrom Macroglobulinemia

## Abstract

Bing‐Neel syndrome (BNS) remains a rare complication of Waldenstrom Macroglobulinemia. Given the paucity of this disease, treatment guidelines are based on small clinical trials with limited participants. Here, we present a case of primary CNS diffuse large B‐cell lymphoma masqueraded as BNS that developed while on ibrutinib therapy.

## Graphical abstract

Even when disease appears stable while on systemic therapy for Waldenstrom Macroglobulinemia, CNS complications may arise, and early recognition can lead to improved quality of life and progression‐free survival during these rare instances.

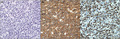

## 1 INTRODUCTION

Waldenstrom macroglobulinemia (WM) is a form of lymphoplasmacytic lymphoma characterized by the malignant proliferation of monoclonal immunoglobulin M (IgM)‐producing B‐lymphocytes resulting in a wide spectrum of complications.^1^ WM is a rare disorder with an estimated incidence rate of 3 per 10^6^ new cases per year.^2^ Although presenting signs and symptoms of this disease can vary greatly from patient to patient, all are related to bone marrow infiltration of lymphoplasmacytic cells as well as the effects from a monoclonal gammopathy causing hyperviscosity, end‐organ deposition, and autoimmune disease.^1^, ^2^ Clinical manifestations include constitutional symptoms such as fevers, night sweats, and unintentional weight loss, as well as symptoms related to anemia and hyperviscosity such as fatigue, dyspnea, and headaches.^1^, ^2^ Neurological complications associated with hyperviscosity from WM include visual changes (ie, blurred vision), vertigo, tinnitus, and peripheral neuropathy, which is most commonly characterized by bilateral and symmetrical sensory deficits in the hands and feet that can progress to difficulty writing and gait instability.^1^, ^2^ Disease involvement outside of the bone marrow most commonly affects the lymph nodes and spleen, with up to 60% of patients developing extramedullary disease upon relapse.^1^ However, direct invasion of the central nervous system (CNS) with lymphoplasmacytic tumor cells, referred to as Bing‐Neel syndrome (BNS), remains an extremely rare complication of WM affecting less than 1% of cases.^2^, ^3^ Bing‐Neel syndrome usually presents as a feature of relapsing disease and should be suspected when there is the onset of central neurological deficits such as altered mentation, cranial nerve deficits, seizure‐like activity, gait disturbances, or even psychiatric disease.^2^, ^3^ Symptoms are usually gradual, progressing over weeks to months, and may be mistaken as hyperviscosity syndrome or neuropathy.^3^ It can be difficult to assess whether new neurological findings, including new brain lesions, represent BNS or possibly the development of a separate primary CNS lymphoma. Here, we present a case of WM with malignant CNS involvement visualized on MRI raising concern for BNS, but with biopsy results revealing diffuse large B‐cell lymphoma (DLBCL).

## 2 CASE PRESENTATION

**FIGURE 1:**
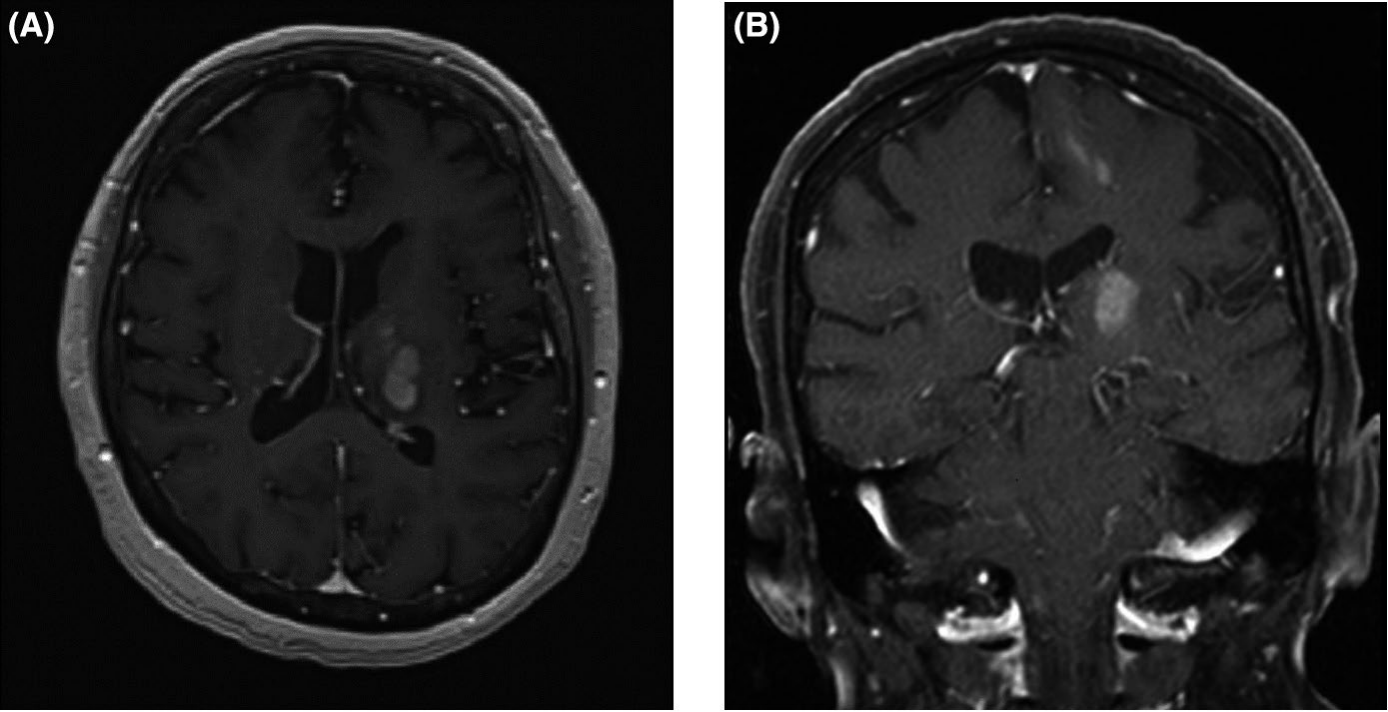
T1‐weighted post‐contrast magnetic resonance imaging (MRI) of the brain obtained when the patient was initially diagnosed with Bing‐Neel Syndrome. An expansile mass with associated heterogeneous internal enhancement is observed within the left thalamus measuring 3.3 × 2.5 cm

A 70‐year‐old Caucasian woman with medical history significant for stage III chronic kidney disease, transitional cell ureteral cancer status post‐left‐sided nephroureterectomy, and three‐year history of Waldenstrom's macroglobulinemia (WM) presented with complaints of right‐sided weakness associated with paresthesias, dysarthria, and blurry vision of three weeks duration. Magnetic resonance (MRI) imaging of the brain demonstrated an enhancing, hypercellular mass centered in the left thalamus with additional foci of signal abnormality and enhancement in the cortex of the left frontal lobe and subcortical white matter (Figure 1). These findings were concerning for an intracranial neoplastic process, especially given her history of WM.

Regarding her oncological history, she was initially diagnosed with WM at the age of 67 after workup for complaints of chronic fatigue revealed elevated IgM levels (3370 mg/dl) as well as serum hyperviscosity. Bone marrow biopsy showed a low‐grade B‐cell lymphoma with plasmacytic differentiation and 60%–70% bone marrow involvement. Neoplastic cells were found to be lambda restricted and negative for CD5, CD10, and CD23 by flow cytometry. An increased number of lambda predominant cells were confirmed by flow cytometry and CD138 immunostaining. The patient was started on first‐line therapy with the Bruton tyrosine kinase inhibitor ibrutinib; however, due to worsening adverse effects after 6 months of therapy she transitioned to rituximab, an anti‐CD20 monoclonal antibody. Unfortunately, the patient was found to have worsening IgM levels and serum viscosity while on rituximab monotherapy over the next 6 months. Thus, she was restarted on ibrutinib while continuing rituximab every 3 months and had significant improvement on this combination of therapy.

**FIGURE 2:**
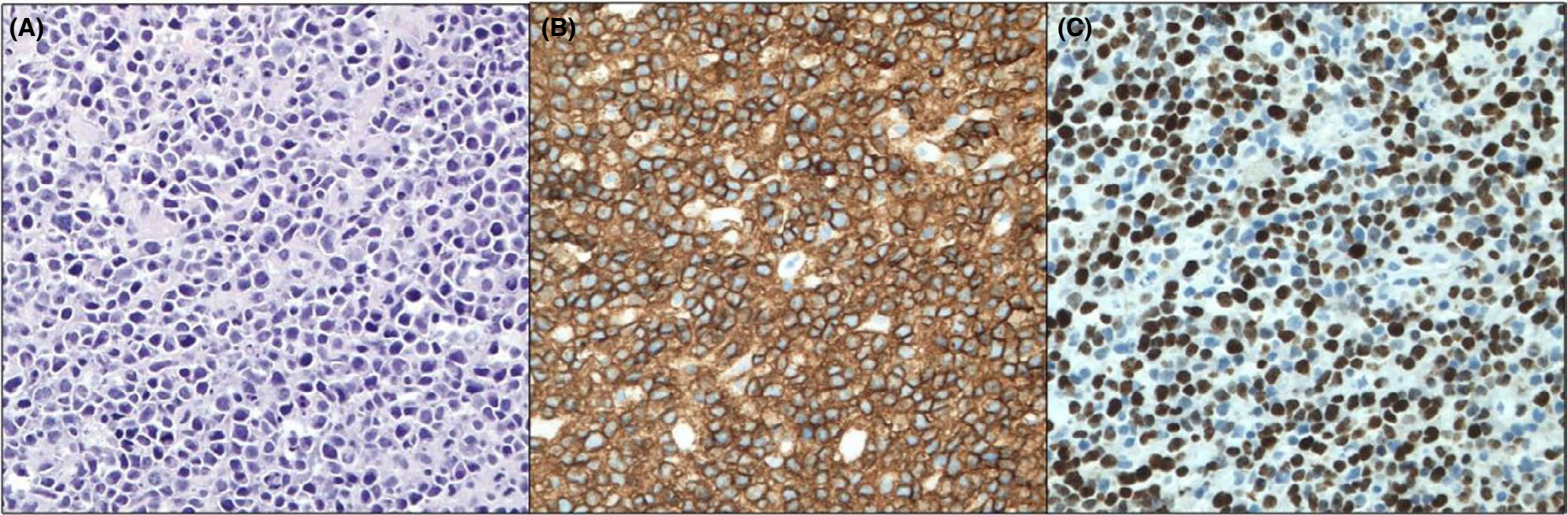
Histology and immunophenotype of Diffuse Large B‐cell Lymphoma. Diffuse mononuclearcell infiltrate, which comprises medium size to large cells (A, H&E ×200). Abnormal cells showing positive expression for the B‐cell antigen CD20 (B, ×200). High proliferation fraction demonstrated by the Ki67 antigen (C, ×200)

She completed two years of maintenance rituximab and reduced‐dose ibrutinib (140 mg) at time of presentation with the most recent IgM levels of 299 mg/dl prior to the onset of her previously mentioned neurological symptoms. Given her MRI findings, computed tomography (CT) imaging of the head, chest, abdomen, and pelvis was completed, which revealed multiple intracranial lesions but no evidence of lymphadenopathy or neoplastic process elsewhere. She further underwent lumbar puncture for cerebral spinal fluid (CSF) analysis with flow cytometry showing mostly T cells without evidence of B‐cell non‐Hodgkin lymphoma. As there remained high suspicion for central nervous system (CNS) lymphoma, the patient ultimately had a left parietal stereotactic brain biopsy with pathology findings of diffuse aggressive B‐cell non‐Hodgkin lymphoma (Figure 2A). Immunohistochemical studies were positive for CD20, CD23, BCL‐6, MUM1, and LE1 (Figure 2B) with approximately 80% of cells expressing Ki‐67 proliferation antigen (Figure 2C). Fluorescent in situ hybridization (FISH) analysis was negative for c‐MYC, BCL‐6, or BCL2 gene rearrangements. Lastly, mutation testing using next‐generation sequencing returned positive for MYD88 L265P mutation.

**FIGURE 3:**
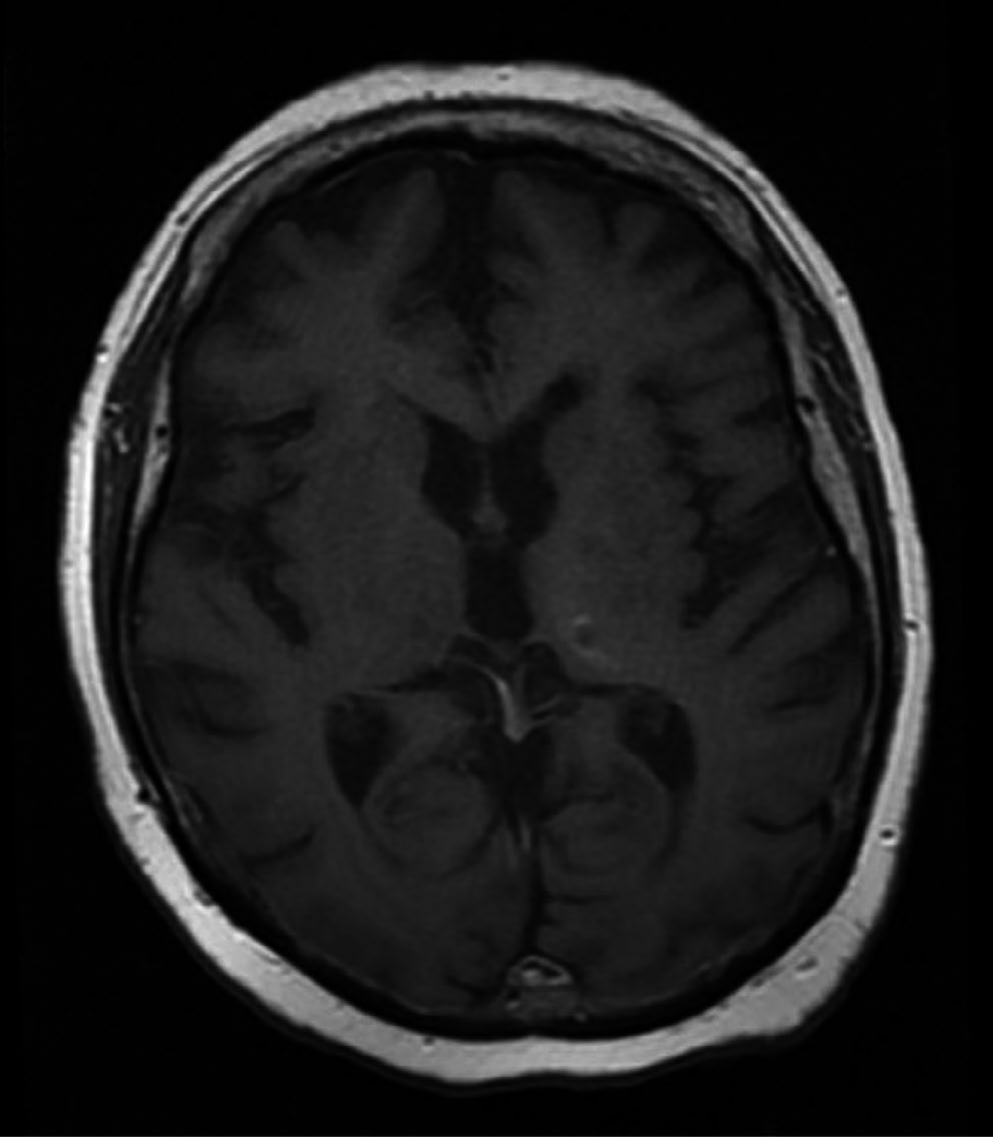
Restaging T1‐weighted magnetic resonance imaging (MRI) of the brain following 2 months of whole brain radiation therapy (WBRT) to 30.6 Gy while continuing systemic treatment with ibrutinib for diagnosed Bing‐Neel Syndrome

The patient was placed on oral dexamethasone 4 mg four times daily with noticeable improvement in her speech and mobility. Due to the patient's poor renal function, she was not a candidate for induction therapy with methotrexate. Thus, she began treatment with whole brain radiation therapy (WBRT) to 30.6 Gy while continuing systemic treatment with ibrutinib. A repeat MRI of the brain two months later demonstrated near resolution of the patient's lymphoma with findings of only a few small foci of nonspecific enhancement adjacent to the biopsy cavity within the left thalamus (Figure 3). There was no evidence of intracranial mass effect, midline shift, or abnormal extra‐axial collection.

## 3 DISCUSSION

Waldenstrom macroglobulinemia (WM) is defined by the World Health Organization (WHO) as an indolent lymphoplasmacytic lymphoma (LPL) belonging to the category of non‐Hodgkin B‐cell lymphomas (NHL).^4^ Although there remains a lack of specific chromosomal or oncogene abnormalities in LPL, 90% of cases share *MYD88* L265P mutations that are most commonly associated with IgM monoclonal gammopathies such as WM.^4^ While WM remains incurable, median survival rates have increased from 5 to 8 years due to increased awareness of the disease and advancements in therapy over the last decade.^2^ Essential aspects of the initial evaluation for WM include a detailed history and physical examination prior to pursuing diagnostic workup. Although constitutional symptoms of this disease are shared among various other lymphoid malignancies, thorough history taking may reveal clinical manifestations specific to hyperviscosity syndrome such as spontaneous epistaxis, recurrent headaches, visual changes, and blurred vision.^1^, ^2^ Peripherally circulating antibodies not only cause detrimental effects through changes in blood viscosity, but also by their deposition in end‐organs and through immune system autoreactivity.^5^ However, the multiple complications associated with WM are not simply related to the effects of a monoclonal gammopathy. For example, peripheral neuropathy, which is observed in approximately 20% of patients who suffer from this disease process may be a result of direct lymphoplasmacytic infiltration or IgM deposition of nerve fibers, amyloidosis from excess light chain production, or development of autoimmunity.^1^, ^2^

In approximately 1% of WM cases, patients develop Bing‐Neel syndrome (BNS) in which there is infiltration of the CNS by lymphoplasmacytic cells. The absolute incidence of BNS is unknown, but in a retrospective cohort study of 1523 WM patients, only 13 patients (0.8%) were diagnosed with BNS, suggesting a very low prevalence.^3^, ^6^ A review of published literature shows primarily case reports and few small retrospective surveys, demonstrating the rarity of this disease manifestation. Retrospective analysis has demonstrated a significantly lower median survival time (4 months) between symptom onset and diagnosis of BNS as compared to WM.^3^ To further complicate matters, BNS can present at any time during the active treatment course for WM, even when the patient is in apparent remission from disease.^1^, ^2^, ^3^ Rarely, BNS precedes the diagnosis of WM and appears as a primary CNS LPL, having only been described in twenty‐four cases.^7^ Prognosis appears to be better in these cases as compared to those with a prior history of WM whose disease progresses to BNS.^3^

The evaluation for BNS begins with diagnostic testing including gadolinium‐enhanced magnetic resonance imaging (MRI) of the brain and whole spine as well as cerebrospinal fluid (CSF) sampling for cytology, flow cytometry, and mutational analyses.^1^, ^2^, ^3^ Findings on MRI include contrast‐enhanced infiltrations with or without thickening of the meningeal sheaths depending on leptomeningeal involvement, in addition to accentuated diffusion weight imaging (DWI) with elevated or normal apparent diffusion coefficient (ADC) values suggestive of vasogenic edema due to perivascular space invasion by malignant cells.^8^ Of note, there are two categories of BNS that can be distinguished on MRI: a diffuse form, in which there is leptomeningeal and perivascular infiltration versus a tumoral form which is unifocal or multifocal and usually involves the deep subcortical hemispheric regions.^3^, ^8^ Infrequently, infraorbital or periorbital involvement can also be seen.^9^ Although these different forms of BNS from MRI findings are described extensively in literature, actual invasion of malignant lymphoplasmacytic cells may be more extensive than what is evident on imaging. Autopsy reports are scarce given the rarity of this disease, but there appears to be prominent perivascular infiltration by malignant cells without deposition of IgM in BNS that is not readily apparent on MRI imaging.^10^

Despite these sensitive imaging techniques, MRI cannot distinguish between other forms of CNS lymphoma thus necessitating further testing with both CSF analysis and tissue biopsy. CSF should be collected and sent for cytology, flow cytometry, in addition to electrophoresis and immunofixation in order to determine whether M‐protein and specific immunoglobulin elevations are present within the CNS.^1^, ^2^, ^3^ Yet, results from immunofixation may be skewed by other form of lymphoplasmacytic lymphomas. Thus, mutational analysis of CSF plays a prominent role in the workup of BNS. As is seen in approximately 90%–97% of WM cases, MYD88 L265P gene mutation identified by either next‐generation sequencing (NGS) or real‐time quantitative PCR (qPCR) aids in diagnosis of BNS and the absence of such may be associated with poorer prognosis.^3^, ^11^, ^12^, ^13^ As demonstrated by Hiemcke‐Jiwa et al, when clinical suspicion remains high for BNS and there is concern for low tumor DNA concentration within the CSF, highly sensitive double droplet PCR (ddPCR) techniques may be used as an alternative for identifying MYD88 L265P mutations.^12^ In addition, testing for immunoglobulin gene rearrangements can help establish whether or not there are monoclonal heavy and light chain gene rearrangements in lymphoplasmacytic cells identified from the CSF, further augmenting the diagnostic workup of BNS.^3^ Of note, the presence of these genetic mutations in either biopsy or CSF analysis is not necessarily specific for BNS as it can be found in other forms of primary CNS lymphoma.^3^, ^11^, ^12^ Regardless of CSF results, biopsy of cerebral or meningeal lesions remains the gold stand for diagnosis of BNS with pathological evidence of lymphoplasmacytic lymphoma and immunochemical demonstration of monotypic B cells expressing antigens such as CD19, CD20, CD79a, and CD79b.^3^

Given the paucity of BNS cases, there remains a lack of universal guidelines pertaining to the management of this disease process. Although there is no standardized approach for treatment, therapy should begin with identifying individuals who are symptomatic from their disease and requires antineoplastic agents that have the ability to penetrate the blood‐brain barrier or agents available for intrathecal administration.^3^, ^14^, ^15^ Treating those who are asymptomatic is felt to be contraindicated since the aim of treatment is not curative and tailored more toward reversing symptoms and inducing long term progression free survival.^3^, ^14^ Previously, systemic chemotherapy with purine nucleoside analogs such as cytarabine, fludarabine, and bendamustine, known to be effective at treating other lymphoproliferative disorders such as WM, showed promise as therapy for the treatment of BNS.^16^, ^17^, ^18^ High‐dose methotrexate and rituximab have been effective for the treatment of BNS as well, but only in combination with one another, as monotherapy with either of these agents has proven to be ineffective.^14^, ^16^ Rituximab itself is believed to have poor penetrance of the blood‐brain barrier, thus contributing to its modest effect. In recent years, the oral Bruton tyrosine kinase (BTK) inhibitor ibrutinib has demonstrated promising results for the treatment of BNS, with it already having proven to be effective as therapy for WM.^19^, ^20^, ^21^ The CNS penetrance of ibrutinib has been well‐established in mouse models in addition to individual cases assessing the concentrations of active metabolite, PCI‐45227, between synchronous measurements of plasma and CSF.^20^ In a multicenter study conducted by Castillo et al. involving 28 patients with BNS treated with ibrutinib, it was found that 86% of patients had improvement of their associated symptoms with 83% of cases demonstrating tumor response on brain MRI.^19^

Additional potentially targetable mutations have been identified throughout the past decade and thus offer further therapeutic alternatives for the treatment of WM and BNS. WHIM (warts, hypogammaglobulinemia, infections, and myelokathexis) syndrome caused by heterozygous mutations within the CXCR4 gene is observed in the pediatric population and characterized by chronic noncyclic neutropenia.^22^ Although MYD88 L265P mutation is the most common somatic mutation in WM, this is followed closely by CXCR4 WHIM‐like frameshift and nonsense mutations.^23^, ^24^ Plerixafor is an FDA‐approved CXCR4 partial agonist and allosteric antagonist of CXCR7, which has been studied for clinical efficacy and safety in treatment of WHIM syndrome.^25^, ^26^, ^27^ In vitro studies have demonstrated that ibrutinib resistance can be potentially reversed by CXCR4 inhibition, yet MYD88 inhibition superseded the survival benefits provided by CXCR4 frameshift mutations.^28^ Therefore, it is theorized that plerixafor may have potential use in reversing ibrutinib failure in patients with WM and its efficacy is currently being evaluated within clinical trials. CXCL12 is another molecule that has been identified as an activator of AKT 1 and MPK1 pathways, which helps confer the ability of malignant WM cells to resist ibrutinib‐triggered apoptosis through mechanisms that increase frameshift and nonsense mutations.^26^ Through continued research of molecular alterations leading to the development of WM and BNS, as well as genetic pathways contributing to resistance of targeted therapy, we can expect to see further improvement in both progression free and overall survival in this patient population.

Our case illustrates the struggle clinician's face when attempting to differentiate between primary CNS lymphoma versus progression to BNS in patients with previously established WM. In our patient's situation, switching from ibrutinib to rituximab had short‐lived efficacy and soon led to worsening serum viscosity and IgM gammopathy. It is possible that she developed CNS signs and symptoms at the time of disease progression from an inadequate response to rituximab monotherapy because of its poor blood‐brain barrier permeability. It is unclear whether or not the patient's other medical conditions including stage III chronic kidney disease and previously treated transitional cell ureteral cancer conferred an increased risk for CNS disease. Resuming therapy with ibrutinib resulted in significant improvements of her lab values and symptoms. However, despite continued treatment and relatively stable disease the patient developed neurological complications and was found to have CNS lymphoma. Diagnostic studies revealed diffuse large B‐cell lymphoma raising suspicion for either the development of primary CNS lymphoma versus progression of WM to BNS. Because large cell transformation in WM is a rare occurrence, it is likely that this case represents primary CNS lymphoma masquerading as BNS. The patient's DLBCL demonstrated a MYD88L265P mutation that is found in both the lymphoplasmacytic cells of WM as well as primary CNS lymphomas. Therefore, clonality studies between bone marrow biopsy and brain tissue are required in such cases in order to confirm a diagnosis of BNS. Unfortunately, bone marrow biopsy was not performed when our patient presented with neurological complications, thus the percentage of lymphoplasmacytic cell infiltration was also unknown at the time. In hindsight, ibrutinib resistance demonstrated through a C481S BTK mutation from the patient's brain biopsy would have further aided in the evaluation for BNS in the event that the patient's WM became resistant to treatment. Once systemic therapy was combined with WBRT there was noted improvement in both her neurological symptoms as well as extent of disease evident on repeat imaging. We believe this case outlines the importance of recognizing early signs and symptoms of CNS involvement within those who have a history of WM, regardless of their current and past treatment regimens. Diagnostic evaluation should be thorough and include biopsy of both intracranial lesions as well as the bone marrow for clonality study comparison when suspicion remains high for BNS in order for there to be clear differentiation between progressions of WM versus development of a primary CNS lymphoma. Early recognition of these disease processes can ultimately lead to more prompt treatment and rapid improvement of symptom burden, extended progression free survival, and overall improved quality of life in this patient population.

## CONFLICT OF INTEREST

The authors of this manuscript declare no conflict of interest.

## AUTHOR CONTRIBUTIONS

Lukas Delasos: contributed to the preparation and presentation of the published work through creation of the initial draft, review of literature, and development of the finalized manuscript. Deep Phachu and Nishka Shetty: contributed to critical review, commentary, and revision of the manuscript. Melissa Sepulveda‐Ramos: contributed to the pathological images and analysis. James Vredenburgh: contributed to critical review and final revisions to the manuscript.

## ETHICAL APPROVAL

This manuscript was completed in accordance with the ethical standards of the institutional research committee.

## CONSENT

The authors have confirmed during submission that patient consent has been signed and collected in accordance with the journal's patient consent policy.

## Data Availability

The data that support the findings of this study are available on request from the corresponding author. The data are not publicly available due to privacy or ethical restrictions.
